# Molecules Present in Plant Essential Oils for Prevention and Treatment of Colorectal Cancer (CRC)

**DOI:** 10.3390/molecules26040885

**Published:** 2021-02-08

**Authors:** Giovannamaria Petrocelli, Fulvia Farabegoli, Maria Chiara Valerii, Catia Giovannini, Alberto Sardo, Enzo Spisni

**Affiliations:** 1 Department of Biological, Geological and Environmental Sciences, University of Bologna, Via Selmi 3, 40126 Bologna, Italy; giovannam.petrocell2@unibo.it; 2 Department of Pharmacy and Biotechnology (FaBiT), University of Bologna, Via San Giacomo 14, 40126 Bologna, Italy; fulvia.farabegoli@unibo.it; 3 Department of Medical and Surgical Sciences, University of Bologna, Via Massarenti 9, 40138 Bologna, Italy; chiaravalerii@hotmail.it; 4 Centre for Applied Biomedical Research—CRBA, St. Orsola-Malpighi University Hospital, Via Massarenti 9, 40138 Bologna, Italy; catia.giovannini4@unibo.it; 5 Department Experimental, Dignostic and Specialty Medicine (DIMES), University of Bologna, Via Massarenti 9, 40138 Bologna, Italy; 6 Xeda International SA, Zone Artisanale la Crau, RD 7, 13670 Saint-Andiol, France; albertos@xeda.com

**Keywords:** essential oils, bowel, colorectal cancer, cinnamaldehyde, eugenol

## Abstract

Essential oils (EOs) are a complex mixture of hydrophobic and volatile compounds synthesized from aromatic plants, commonly present in the human diet. In recent years, many in vitro studies have suggested possible anticancer properties of single EO compounds, on colorectal cancer (CRC) cells. However, the majority of these studies did not compare the effects of these compounds on normal and cancer colon cells. By using NCM-460, a normal human mucosal epithelial cell line, Caco-2, a human colon epithelial adenocarcinoma cell line, and SW-620, colon cancer cells derived from lymph node metastatic site, we identified cinnamaldehyde, derived from cinnamon EO and eugenol, derived from bud clove EO, as compounds with a specific anticancer action selectively targeting the transformed colonic cells. Both cinnamaldehyde (75 µM) and eugenol (800 µM), after 72 h of treatment, were capable to induce apoptosis, necrosis and a cell cycle slowdown in Caco-2 and in SW-620, but not in NCM-460 cells. If associated with a targeted delivery to the colon, these two compounds could prove effective in the prevention or treatment of CRC.

## 1. Introduction

Essential oils (EOs) are complex mixtures of volatile compounds produced by plants as secondary metabolites. They are highly volatile, liquid and soluble in lipids and organic solvents [1]. Usually, EOs are extracted using hydrodistillation with boiling water or steam [2,3]. Bioactive compounds, such as monoterpenes, sesquiterpenes and aromatic molecules, can be found in different proportions in EOs and characterize their biological properties [4]. These properties include antibacterial [5,6], antioxidant [7,8] and anti-inflammatory activity, which makes them very interesting to chemoprevention aims because chronic inflammation is often linked to carcinogenesis [9,10,11,12]. 

**Figure 1:**
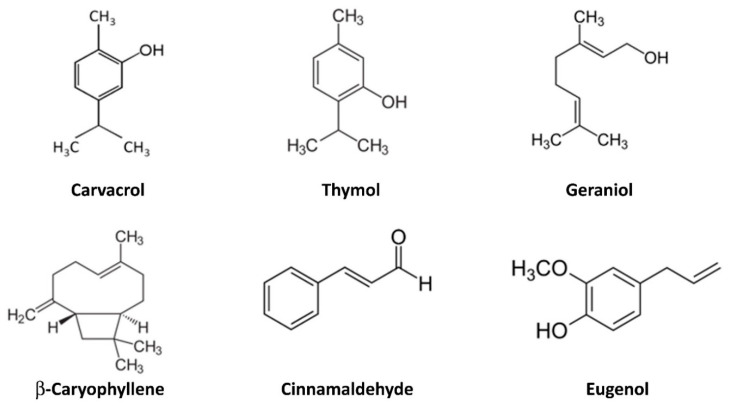
Chemical structure of the chosen molecules present in essential oils whose selective antitumor activity has been studied on colonocytes. Note that carvacrol and thymol are structural isomers.

Colorectal carcinoma (CRC), which ranks second in terms of mortality and third in terms of incidence worldwide, develops gradually over the years as a result of genetic and epigenetic modifications [13]. Gene mutations, polymorphism of several proteins, chromosomal instability, genome global hypermethylation, environmental factors (alcohol, diet, smoke), gut microbiota (production of detrimental factors, increase in noxious bacteria levels) and chronic intestinal inflammation, such as inflammatory bowel disease (IBD), all raises the risk of CRC development [14]. In particular, the rising incidence of IBD is exposing a larger number of individuals to a higher risk of CRC development in the future [15,16]. An increased risk of CRC development is also related to a group of pre-neoplastic conditions, such as familial adenomatous polyposis (FAP), Peutz-Jeghers syndrome, Lynch syndrome and serrated polyposis syndrome. In these cases, to delay or eradicate the development of CRC, a preventive approach should be adopted. Despite the new advances in CRC therapy [15], prevention strategies are still an important tool for public health, as lifestyle and, in particular, eating habits are considered a risk factor among the populations of the western countries. From this point of view, EO compounds could represent a smart strategy to prevent carcinogenic lesions, which are often associated with chronic bowel inflammation, and which are difficult to detect with the standard diagnostic tools in use [16]. Given the advances in primary and adjuvant treatments, the survival of CRC patients has been improved. However, despite the emergence of numerous screening programs a quarter of CRCs are diagnosed at an advanced stage, which is difficult to treat with a surgical approach. For those patients with unresectable lesions, the goal is to avoid continuous tumor spread and growth, and radiotherapy and chemotherapy are the leading strategies for controlling disease in such patients where the use of EO compounds alone or in combination with conventional chemotherapeutic strategies should be explored. To define whether EO compounds might be suitable in advanced CRC treatment, the lack of toxicity on normal cells of human colon mucosae ought to be assessed. We decided to focus on six EO molecules: carvacrol, thymol, geraniol, β-caryophyllene, cinnamaldehyde and eugenol (Figure 1). All these molecules have been demonstrated to be toxic on numerous human cancer cells, including CRC cells [17], whereas data concerning their toxicity on normal human colon epithelial cells are lacking. Thus, we have tested the effects of EO compounds on both NCM-460, a normal human mucosal epithelial cell line, Caco-2, a human colon epithelial adenocarcinoma cell line, and SW-620, a colon cancer cell line derived from a lymph node metastatic site. Interestingly, we describe for the first time that some EO molecules induce apoptosis and cell cycle arrest in CRC cell lines while having no effect on normal cells, suggesting that they should be used in combination with conventional cancer therapy without damaging normal epithelial cells and deserve further clinical exploration.

## 2. Results

### 2.1. Identification of Single Components with Selective Toxicity for CRC Cell Lines

Our first question was whether a single administration of EO compounds plays any effect on cells vitality. Thus, we performed a preliminary screening of two CRC cell lines (Caco-2 and SW-620) and a normal mucosal epithelial cell line (NCM-460) by MTT assay. The cytotoxic effects of carvacrol, geraniol, β-caryophyllene, eugenol, thymol and cinnamaldehyde were first evaluated on NCM-460 and Caco-2 cell lines by using MTT assay. A range of concentrations (five or six concentrations) was tested for each single component, based on the literature. The second time, we also tested three relevant concentrations on the SW-620 cell line, to find out a possible cytotoxic effect even on metastatic cells.

Carvacrol was tested in a concentration range of 150–1000 µM. At low doses, it already exhibited a strong toxicity after 72 h, especially in the normal cell line. The differences were statistically significant. Then, we also tested carvacrol at 250, 500 and 750 µM concentrations on SW-620 cell line, which resulted in a modest statistically significant toxicity only after 72 h (Figure S1). At all the concentrations tested, the cytotoxic effect of carvacrol was similar or greater in NCM-460 than in tumor cells.

Geraniol (100–800 µM) did not show a cytotoxic effect on NCM-460, Caco-2 and SW-620 cells. On the contrary, it seemed to increase cell proliferation (NCM-460 at 48–72 h and Caco-2 at 72 h). Only at the highest concentration (1000 µM), there was a strong toxicity on the NCM-460 and SW-620 cell lines after 72 h treatment, but not on the Caco-2 cells (Figure S2).

β-caryophyllene at low doses (120–240 µM) affected the viability of NCM-460 (240 and 360 µM at 24 h and 120–260 µM at 72 h) and SW-620 cells (260 µM at 24, 48 and 72 h). In contrast, increased cell growth was detected in Caco-2 cells at 120 and 240 M. All the three cell lines showed a significant cytotoxicity after 360 and 480 µM β-caryophyllene treatments for 72 h (Figure S3).

Thymol (concentrations ranging from 165 to 1650 µM) was cytotoxic to NCM-460 cells at all the tested concentrations at any timepoint. The cytotoxic effect was also detectable on Caco-2 cell lines (from 330 to 1650 µM at any timepoint) as well as in SW-620 cells at 660 and 1320 µM (Figure S4).

**Figure 2:**
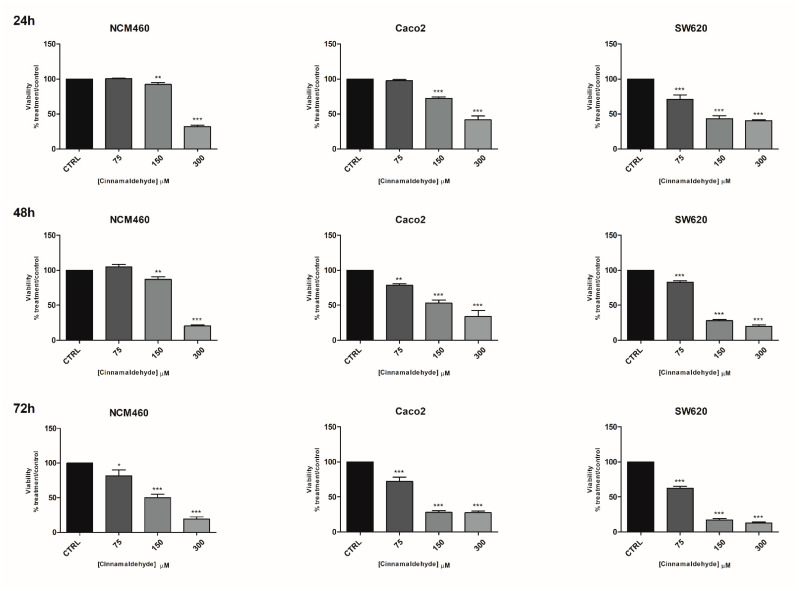
Effects of cinnamaldehyde on the viability of NCM460, Caco2 and SW620 cell lines. Effects of cinnamaldehyde on the viability of NCM460, Caco2 and SW620 cell lines at three timepoints: 24, 48, 72 h evaluated by MTT. CTRL: control untreated cells; NCM460: human normal colon mucosal epithelial cells; Caco2: human epithelial colorectal adenocarcinoma cells; SW620: colon cancer cells derived from lymph node metastatic site. The values were normalized to the untreated controls. The results are expressed as average ± SE of three independent experiments. Significant statistical differences were evaluated with one-way ANOVA, followed by Bonferroni’s Multiple Comparison test: * *p* < 0.05; ** *p* < 0.01; *** *p* < 0.001.

Cinnamaldehyde was tested in a concentration range 40–600 µM (Figure S5) on NCM-460 and Caco-2 cells. The data showed a high statistically significant cytotoxic effect at any timepoint after 300 µM treatments. Thus, the two highest concentrations (450 and 600 µM) were excluded in the following experiments. A modest, but statistically significant, cytotoxic effect was detected in NCM-460 cells after 75 µg/mL treatment for 72 h (Figure 2). This concentration reduced viability of Caco-2 cells after 48 and 72 h treatments and SW-620 cells at any timepoint. It is noteworthy that cinnamaldehyde cytotoxic effect was greater in SW-620 than in Caco-2 cells. As regards the IC_50_, at 24 h, they were 218, 166 and 92 µM, respectively, for the NCM-460, Caco-2 and SW-620 cells, while at 72 h they were very similar—respectively, 83, 87 and 92 µM for the NCM-460, Caco-2 and SW-620 cells (Table S1). Thus, while for cinnamaldehyde the selectivity index was 1.31 for the Caco-2 and 2.27 for the SW-620 at 24 h, this selectivity completely disappeared after 72 h (Table S1).

**Figure 3:**
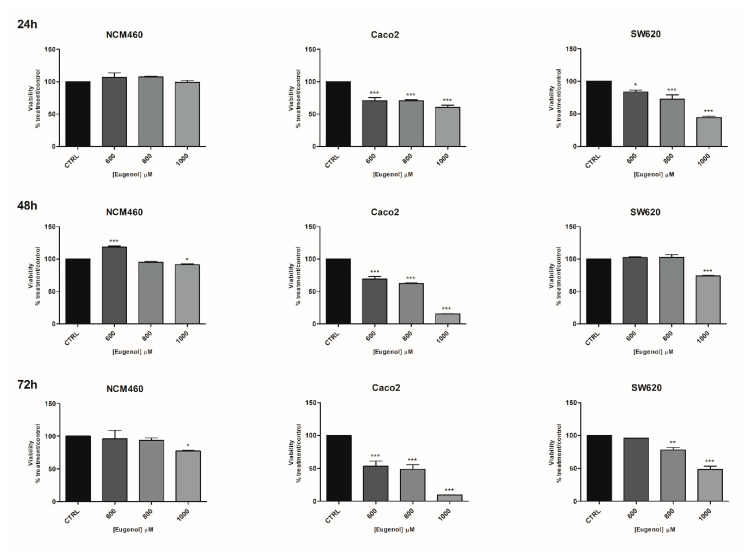
Effects of eugenol on the viability of NCM460, Caco2 and SW620 cell lines. Effects of eugenol on the viability of NCM460, Caco2 and SW620 cell lines at three timepoints: 24, 48, 72 h. CTRL: control untreated cells; NCM460: human normal colon mucosal epithelial cells; Caco2: human epithelial colorectal adenocarcinoma cells; SW620: colon cancer cells derived from lymph node metastatic site. The values were normalized to the untreated controls. The results are expressed as average ± SE of three independent experiments. Significant statistical differences were evaluated with one-way ANOVA, followed by Bonferroni’s Multiple Comparison test: * *p* < 0.05; ** *p* < 0.01; *** *p* < 0.001.

A broad range of concentrations of eugenol (100–1000 µM) was first used on NCM-460 and Caco-2 cells (Figure S6). The lower doses (from 100 to 400 µM) seemed to stimulate NCM-460 and Caco-2 cell line proliferation. Thus, higher concentrations were used to investigate the effect of eugenol on the three cell lines used in the study. A significant cytotoxic effect of eugenol at 800 and 1000 µM was detected on Caco-2 and SW-620 cell lines starting from 24 h treatment (Figure 3) with respect to NCM-460 normal cells. Only a modest cytotoxic effect on NCM-460 cells also occurred after 48 and 72 h at the highest doses tested (1000 µM). Overall, eugenol toxicity was more effective on Caco-2 than SW-620 cells and its toxicity on NCM-460 was very scarce. As regards the eugenol IC_50_, at 24 h, they were 1371, 458 and 918 µM, respectively, for the NCM-460, Caco-2 and SW-620 cells, while at 72 h they were 1161, 730 and 866 µM, respectively, for the NCM-460, Caco-2 and SW-620 cells (Table S1). Thus, for eugenol, the selectivity index was 2.82 for the Caco-2 and 1.49 for the SW-620 at 24 h, and this selectivity was maintained at 72 h: 1.60 for the Caco-2 and 1.34 for the SW-620 (Table S1).

On the bases of the results obtained, we focused on eugenol and cinnamaldehyde, which showed higher cytotoxicity on Caco-2 and SW-620 cells with respect to the normal cells.

### 2.2. Cell Cycle Analysis

**Figure 4:**
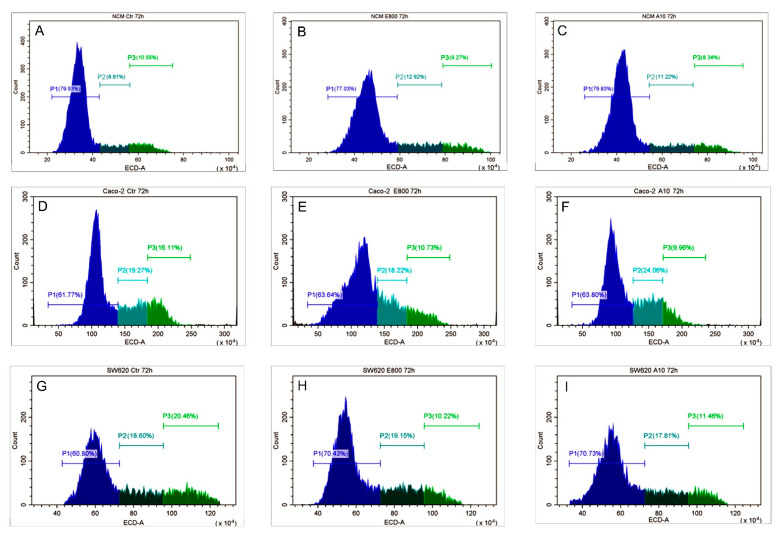
Cell cycle analysis. Evaluation of cell cycle of NCM460 (**A**–**C**), Caco2 (**D**–**F**), and SW620 (**G**–**I**) after 72 h treatment with eugenol 800 µM (E800) and cinnamaldehyde 75 µM (A75). P1 (pre-G1/G1 phase); P2 (S phase); P3 (G2/M phase). The results shown are representative of 3 different experiments.

The reduction in cell growth observed with the MTT assay moved us to investigate how cell cycle could be affected by EO compounds. For the cell cycle analysis, we chose the lowest concentrations of eugenol and cinnamaldehyde, which gave an effect on both CRC cell lines. Thus, the effects of 75 µM cinnamaldehyde and 800 µM eugenol on the cell cycle progression were evaluated 72 h post-treatment. Concerning NCM-460 cells, we did not find differences between the treated (both cinnamaldehyde and eugenol) and control samples (Figure 4A–C). In contrast, we detected a reduction of G2 phase in Caco-2 cells with both treatments (Figure 4D–F). Contrary, in SW-620 cells, cinnamaldehyde and eugenol treatment resulted in G1 accumulation (Figure 4G–I) suggesting that the effects of these molecules on cell cycle could be strongly related to the cell background.

### 2.3. Cell Death Evaluation

**Figure 5:**
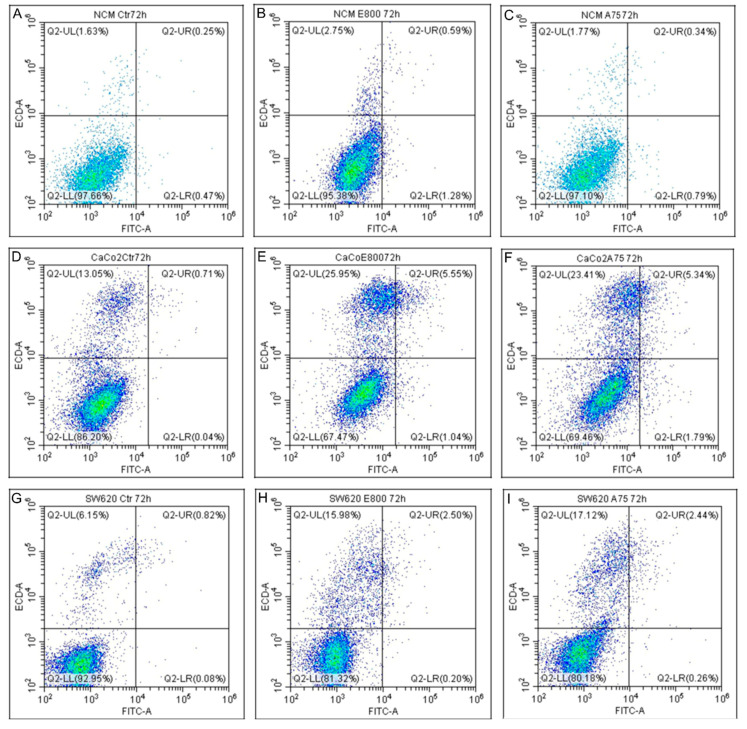
Apoptosis detection. Apoptosis detection by AnnexinV/PI analysis on NCM460 (**A**–**C**), Caco2 (**D**–**F**), SW620 (**G**–**I**). Ctr: control untreated cells. Cells were exposed to treatments with 800 µM eugenol (E800) and 75 µM cinnamaldehyde (A75) for 72 h. Viable cells (lower left, AnnexinV −/PI −), early apoptotic (lower right, AnnexinV +/PI −) and late apoptotic/necrotic cells (upper, AnnexinV +/PI +) quadrant. The results shown are representative of three independent experiments.

To go deeper into the mechanisms sustaining variations of proliferation after EO compounds treatments, cell death was investigated by using Annexin V/PI detection by flow cytometry. The effects of 800 µM eugenol and 75 µM cinnamaldehyde treatments for 72 h were analyzed. Unlike the cancer cells, the treatments did not induce NCM-460 cell apoptosis (Figure 5A–C), whereas both late apoptosis and necrosis were detected in Caco-2 (Figure 5D–F) and SW-620 cells (Figure 5G–I).

Caco-2 cells treated with eugenol showed an increase in late apoptosis (from 0.71% to 5.55%) and necrosis (from 13.05% to 25.95%) (Figure 5D,E). A similar result was detected after cinnamaldehyde treatment (Figure 5F). Necrosis increase was indeed the most remarkable finding in SW-620 cells after both cinnamaldehyde and eugenol treatments (15.98% and 17.12%, respectively, in comparison to 6.15% in control cells) (Figure 5G–I).

## 3. Discussion

EO compounds have been found to exert an anticancer activity against numerous human neoplastic cell lines, including CRC, either alone or in association with anticancer drugs [18]. However, there is little evidence about the effects of these compounds on normal cell lines, especially normal epithelial colonic cells. We speculated that possible EO compounds use for CRC prevention or therapy should be preceded by a toxicological in vitro evaluation of EO molecules on normal cells too. By using the NCM-460 cell line as a normal epithelial colon model, we selected two out of six EO single components (cinnamaldehyde and eugenol) suitable to our aims since they were the best candidates, as per the results, in terms of lack of toxicity on NCM-460 cells and anti-cancer activity towards the CRC cell lines.

The introduction of a normal epithelial colon cell line in the present study was diriment in understanding which EO molecules might be used in CRC prevention and or therapy. Carvacrol and thymol (two major molecules of oregano essential oil) have already been studied on Caco-2 [19] and other tumor cell lines and have been found to be cytotoxic. Herein, we confirmed that carvacrol treatment reduced Caco-2 cell viability in the same range of concentrations used by Llana-Ruiz-Cabello and co-workers [19], but it was ineffective on SW-620 cells and toxic to NCM-460 cells. Accordingly, carvacrol was found to decrease the viability of lymphocytes and intestinal epithelial cells [20]. Thymol was demonstrated to reduce viability of Caco-2 cells and to be not genotoxic (IC_50_ at 700 µM) [21]. In the present study, it decreased viability in Caco-2 and SW-620 cells, but this effect was even greater in NCM-460 cell line. Carvacrol and thymol are structural isomers. Despite this similarity, toxicity data indicate rather different behavior on normal and tumor cells, with thymol showing highest toxic effects on NCM-460 after 24 h, affecting cell viability at 165 μM versus 500 μM of carvacrol. On SW-620 cells, carvacrol showed no toxicity at 24 and 48 h, up to the maximum tested concentration of 750 μM, while thymol was toxic at 660 μM after 24 h. Günes-Bayir and coworkers [22] suggested that the harmful effect of thymol on both normal and cancer cells (human fibroblast and gastric adenocarcinoma) might be related to a hormetic effect.

Geraniol was not cytotoxic to NCM-460 cells, but it showed very modest effects on Caco-2 cell and a significant cytotoxicity on SW-620 cells only at high concentration (1000 μM). These findings agree with previous studies, which reported a low cytotoxic effect after geraniol individual treatment and better results in association with 5-Fluorouracil [22]. β-caryophyllene, a natural compound found in several EOs, notable for having a cyclobutane ring, which is a rarity in nature, affected cell viability but only at high concentrations and in all the three investigated cell lines, and, therefore, it was excluded as a selective anticancer molecule. In agreement with our findings, β-caryophyllene has been found as a cytotoxic compound over a wide range of cell lines even with few selective antiproliferative activity, such as against ovarian cancer cell lines [23].

Cinnamaldehyde and eugenol were found to be effective as anticancer agents in vitro, given individually, or in association with conventional therapy such as Doxorubicin [24,25,26,27,28,29]. To the best of our knowledge, we showed for the first time that these two compounds strongly reduced cell viability of CRC cells with only modest cytotoxic effects on normal cells. The effects of eugenol 800 µM and cinnamaldehyde 75 µM in cells after 72 h of treatment were deeper investigated. A reduction of G2 phase was induced by eugenol and cinnamaldehyde in Caco-2 cells, whereas in SW-620 cells, cinnamaldehyde and eugenol treatment resulted in G1 accumulation. Both molecules did not modify the cell cycle of NCM-460. These results showed that eugenol and cinnamaldehyde in a specific range of concentrations and times can reduce CRC cell proliferation, either by reducing G2 phase or increasing G1 phase of cell cycle, and pointed to the relevance of the molecular background in dictating EO compounds’ effects. Moreover, the Annexin V-FITC evaluation by flow cytometry showed a significant increase in necrosis and late apoptosis in CRC cells treated with eugenol or cinnamaldehyde without effects on NCM-460. The lack of effects in non-tumor cells suggests that using eugenol and cinnamaldehyde for CRC therapies or cancer prevention should be considered and deeply analyzed. The greater cytotoxicity of cinnamaldehyde in SW-620, with respect to Caco-2 cells, indicates a greater selectivity of this molecule for metastatic cells. Unfortunately, this selectivity tends to be lost as the duration of treatment increases.

Indeed, a major limit of the present study was that, while identifying the molecular background as a relevant player mediating the effects of EO compounds, specific drivers were not identified and required further investigation. Di Giacomo and coworkers [29] proposed, for cinnamaldehyde, that non-specific mechanisms, including the alteration of membrane permeability, could be involved in the final effect. Increased permeability in cancer cells, due to a different plasmatic membrane composition, and greater activation of molecular pathways fundamental in cancer cell survival and growth, might explain the selective effect. Another interesting property of cinnamaldehyde and eugenol is that they both show antioxidant and pro-oxidant activities in different contexts. Antioxidant mechanisms have been described as protective against cancer formation (carcinogenesis or tumorigenesis). Once a cancer has developed, the pro-oxidant effects can induce cancer cell death by several signaling pathways [30]. Eugenol was found to induce both pro-oxidant and anti-oxidant effects on human cells, being the pro-oxidant effects prevalent in cancer cells and the anti-oxidant effects in normal cells undergone to oxidative stress [31]. Cinnamaldehyde can also induce the ROS-mediated mitochondrial permeability transition and resultant cytochrome c release and apoptosis [32].

The combination of antioxidant and anti-inflammatory properties of EO compounds is particularly indicated in CRC, as chronic inflammation and oxidation have been demonstrated to promote the neoplastic transformation and progression of colon cancer cells. Furthermore, the relatively long time of transition from a normal colon epithelial cell to a cancer cell in the pre-neoplastic syndromes also offers the opportunity to experiment the use of eugenol and cinnamaldehyde in a proper formulation, to delay and reduce CRC initiation. Nevertheless, the combination of chemotherapeutic drugs and cinnamaldehyde and eugenol, already demonstrated, might also offer a new therapeutic opportunity to improve the anti-cancer effects in advanced CRC types, saving the normal colon epithelial cells.

One of the possible problems in making these compounds effective against CRC development is releasing them selectively and at the right concentrations into the colon. In fact, physiologically, once ingested, they are rapidly absorbed in the small intestine and they often also show short half-lives once reaching the blood circulation, since they are rapidly metabolized by the liver [33]. One solution could be their enema administration which has shown efficacy in murine models [34], while less invasive applications must provide a slow and selective release into the colon, as we have already obtained for geraniol [35,36].

## 4. Materials and Methods

### 4.1. Materials

Geraniol, carvacrol, cinnamaldehyde, eugenol, β-caryophyllene, thymol (natural, ≥97%) were provided by Xeda International S.A. (Saint-Andiol, France). DMEM, penicillin-streptomycin solution, 3-(4,5-Dimethyl-2-thiazolyl)-2,5-diphenyl-2H-tetrazolium bromide (MTT) were purchased from Sigma Aldrich (Milan, Italy). Fetal bovine serum (FBS) was obtained from Microgem. L-glutamine was purchased from Lonza Walkersville INC. DMSO and absolute ethanol were purchased from Carlo Erba (Milan, Italy).

### 4.2. Cell Lines and Culture

NCM-460 cell line was purchased from Bena Culture Collection (Jiangsu, China), Caco-2 and SW-620 cell lines were obtained from the American Type Culture Collection (ATCC) (Manassas, VA, USA). All the cells were cultured in high-glucose DMEM containing 10% FBS, 1% penicillin-streptomycin and 1% L-glutamine at 37 °C in a humidified incubator with 5% CO_2_.

### 4.3. Cell Treatments

Briefly, cells were firstly seeded, in triplicate, into 96-well culture plates at 10^4^ cells/well. After 24 h, medium was replaced with fresh one containing different concentrations of essential oil single components: carvacrol 150, 300, 450, 500, 750, 1000 μM; cinnamaldehyde 37,5, 75, 150, 300, 450, 600 μM; geraniol 100, 200, 400, 600, 800, 1000 μM; eugenol 100, 200, 400, 600, 800, 1000 μM; β-caryophyllene 120, 240, 360, 48, 600 μM; thymol 165, 330, 660, 1320, 1650 μM. Ethanol 99.5% was used to solubilize essential oils. The final concentration of ethanol in the cell medium was kept lower than 0.1%. Ethanol 0.1% was added to the control (untreated) cells.

### 4.4. Cell Viability Assay

The cytotoxicity of the essential oil single components was evaluated by MTT assay on 96-well plates. After 24, 48 and 72 h, MTT (dissolved in PBS) was diluted with fresh medium at final concentration of 0.5 mg/mL, added to cell culture and incubated at 37 °C. After 4 h, the medium was removed and 100 μL of DMSO was added. After 1 h at room temperature, the purple formazan crystals were dissolved, and the plates were read in a microplate reader (Victor3™, Perkin Elmer, Waltham, MA, USA). Absorbance was measured at 595 nm. Cell growth inhibition rate was calculated as a percentage of treated cells on control ones.

### 4.5. Cell Cycle Analysis and Apoptosis Detection Assay

Cell cycle analysis and apoptosis detection assay were only performed in cell treated with cinnamaldehyde and eugenol, at dosages that were not found toxic on the NCM-460 cell line. Briefly, 100,000 cells were seeded into 6-well cultured plates and allowed to attach for 24 h. After that, cells were treated for 72 h as previously described. Then, the cells were collected for the cell cycle analysis, washed twice with PBS, fixed with 70% ethanol, and incubated at −20 °C overnight. Then, the cells were washed with 500 µL of PBS, centrifuged at 1500 rpm for 5 min, resuspended in 500 μL of PBS containing 50 μg/mL RNase A and incubated for 30 min at room temperature. Cells were then centrifuged at 1500 rpm for 5 min, resuspended in PBS, and 10 µg/mL of Propidium iodide was added. Detection of apoptotic cells was performed with Annexin V/Propidium iodide detection kit, following the manufacturer’s instructions (Bender Medsystems, Wien, Austria). Cells were analyzed by flow cytometry (FACS) (Cytoflex, Beckman Coulter, CA, USA).

### 4.6. Statistical Analysis

All data are presented as means ± standard error (SE). Statistical analysis was performed by using Prism 5 (GraphPad, La Jolla, CA, USA). One-way ANOVA with Bonferroni’s Multiple Comparison post-hoc test was applied to compare the treated and the control groups. A *p* < 0.05 value was considered statistically significant.

## 5. Conclusions

Although the mechanisms of action are certainly multitarget and, therefore, not easy to define, it is evident that eugenol and cinnamaldehyde demonstrate a specific antitumor action against CRC cells, within certain dosage and time ranges. Regarding possible relationships between the biological activity and their chemical structure, eugenol is a member of the allylbenzene, while cinnamaldehyde is a phenylpropanoid. Nevertheless, they share chemical-physical characteristics, such as similar molecular mass and solubility, that allow them to easily enter the cell membrane and reach the cytosol and also the nucleus, probably via lipid droplets, thus being able to interfere and modulate many different cellular pathways. With the current state of knowledge, however, it is not possible to predict the prevalent activity of these compounds, within normal or tumor eukaryotic cells, based on their chemical structure, as demonstrated by the different activities of two structural isomers, carvacrol and thymol. So, an experimental approach on cells, animal models or humans is still needed. Further in vivo and clinical studies are necessary to confirm these activities of eugenol and cinnamaldehyde against CRC. Unfortunately, the low cost of these molecules and their non-patentability make them little or not at all interesting to pharmaceutical industries, making sponsorship for clinical studies necessary to finally validate their therapeutic efficacy against CRC.

## Supplementary Materials

The following are available online, Table S1: IC_50_ and Selectivity Index in NCM460, Caco2 and SW620 cells, Figure S1: Effects of carvacrol on the viability of NCM460, Caco2 and SW620 cell lines, Figure S2: Effects of geraniol on the viability of NCM460, Caco2 and SW620 cell lines, Figure S3: Effects of β-caryophyllene on the viability of NCM460, Caco2 and SW620 cell lines, Figure S4: Effects of thymol on the viability of NCM460, Caco2 and SW620 cell lines, Figure S5: Effects of cinnamaldehyde on the viability of NCM460 and Caco2 cell lines, Figure S6: Effects of eugenol on the viability of NCM460 and Caco2 cell lines.

## Sample Availability

Samples of the EO compounds are available from the authors.
