# Supplementary material for: Molecules Present in Plant Essential Oils for Prevention and Treatment of Colorectal Cancer (CRC)
Source: Molecules. 2021 Feb 8;26(4):885. doi: 10.3390/molecules26040885 (PMC7914775; doi:10.3390/molecules26040885)
Supplement: Supplementary file 1 [file molecules-26-00885-s001.pdf]

Supplementary materials

Table S1: IC$_{50}$ and Selectivity Index in NCM460, Caco2 and SW620 cells. IC$_{50}$ expressed in µM was calculated by using GraphPad Prism and the value is reported in the upper part of the Table. Selectivity Index was calculated by dividing the IC$_{50}$ of normal cells (NCM460) for IC$_{50}$ of each neoplastic cell line (Caco2 and SW620). A value > 1 is considered representative of a selective action of the compound in neoplastic versus normal cells.

IC $_{50}$ µM

	Cinnamaldehyde		Eugenol	
	**24 h**	**72 h**	**24 h**	**72 h**
**NCM460**	218.3	83.25	1371	1161
**Caco2**	166.1	86.85	485.3	729.5
**SW620**	91.99	92.11	918.1	866.4

Selectivity Index

	Cinnamaldehyde		Eugenol	
	**24 h**	**72 h**	**24 h**	**72 h**
**Caco2**	1.31	0.96	2.82	1.6
**SW620**	2.37	0.9	1.49	1.34

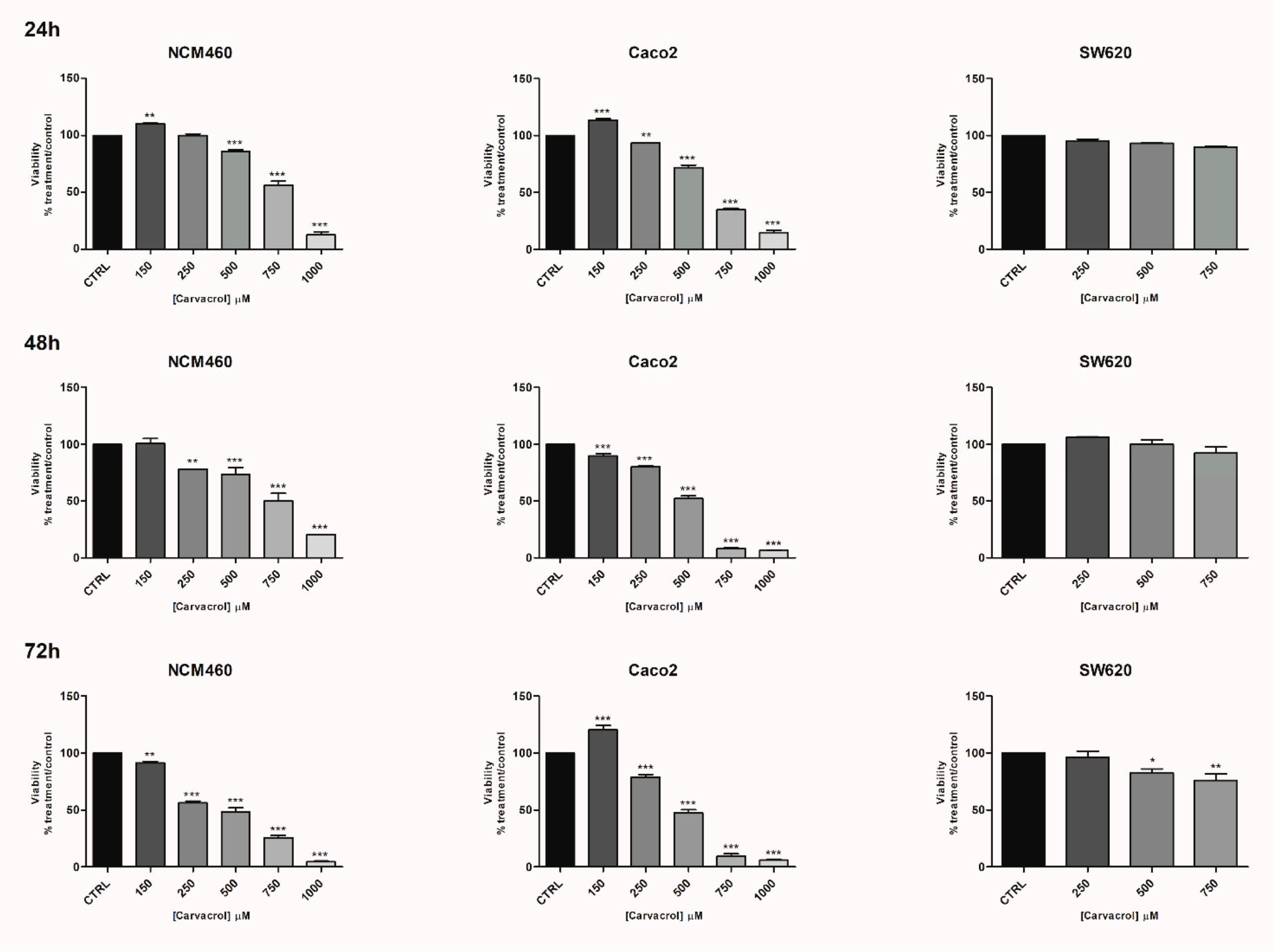

FigureS1. Effects of carvacrol on the viability of NCM460, Caco2 and SW620 cell lines at three time points: 24, 48, 72 hours. The columns show the average median ± SE of three replicate experiments with three samples analysed per replicate. Significant statistical differences were evaluated with one-way ANOVA, followed by Bonferroni's Multiple Comparison test was applied to compare the treatment and the control groups: *=p<0.05; **=p<0.01; ***=p<0.001. NCM460: human normal colon mucosal epithelial cells; Caco2: human epithelial colorectal adenocarcinoma cells; SW620: colon cancer cells derived from lymph node metastatic site.

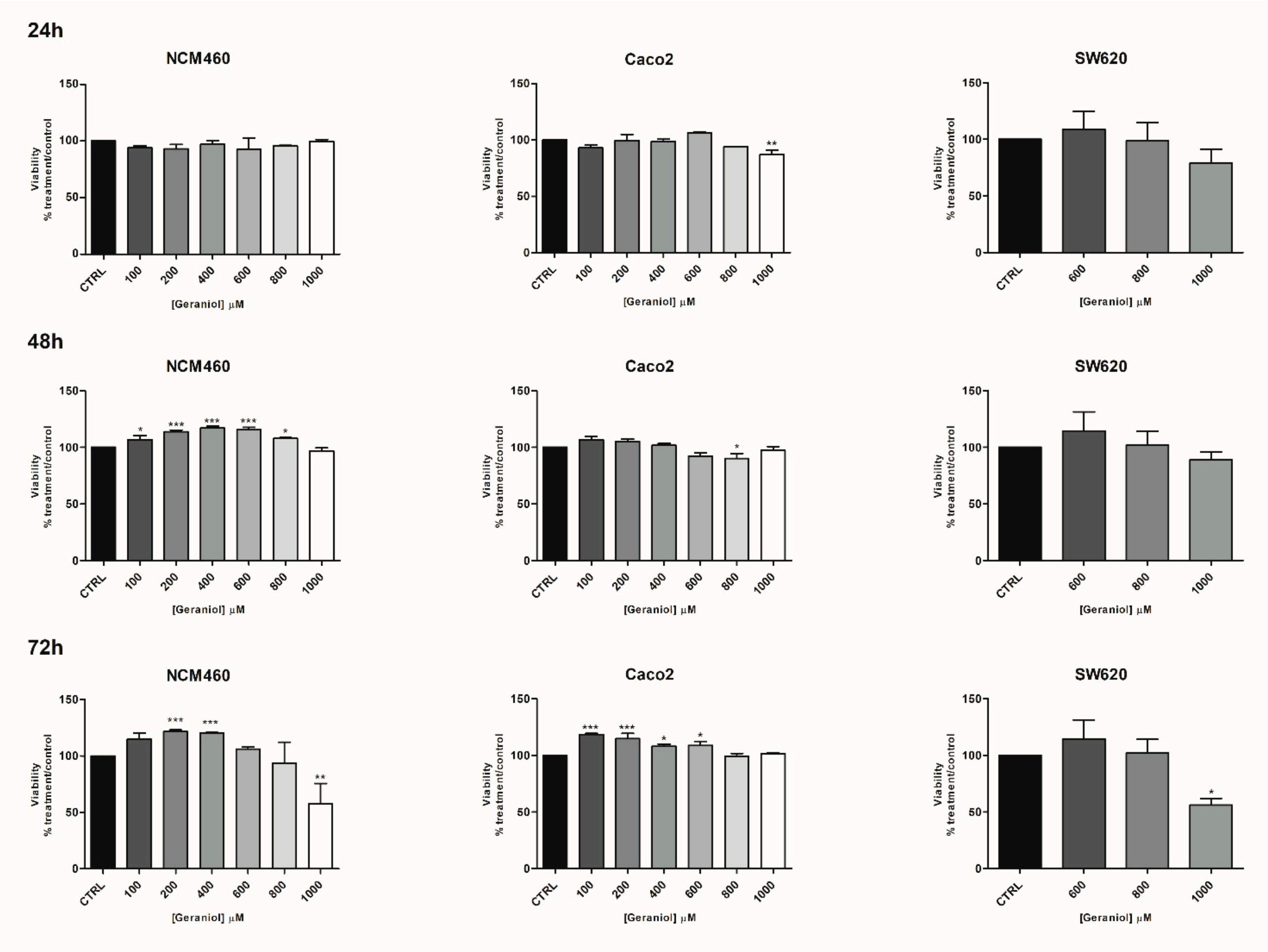

Figure S2. Effects of geraniol on the viability of NCM460, Caco2 and SW620 cell lines at three time points: 24, 48, 72 hours. The columns show the average median ± SE of three replicate experiments with three samples analysed per replicate. Significant statistical differences were evaluated with one-way ANOVA, followed by Bonferroni's Multiple Comparison test was applied to compare the treatment and the control groups: *=p<0.05; **=p<0.01; ***=p<0.001. NCM460: human normal colon mucosal epithelial cells; Caco2: human epithelial colorectal adenocarcinoma cells; SW620: colon cancer cells derived from lymph node metastatic site.

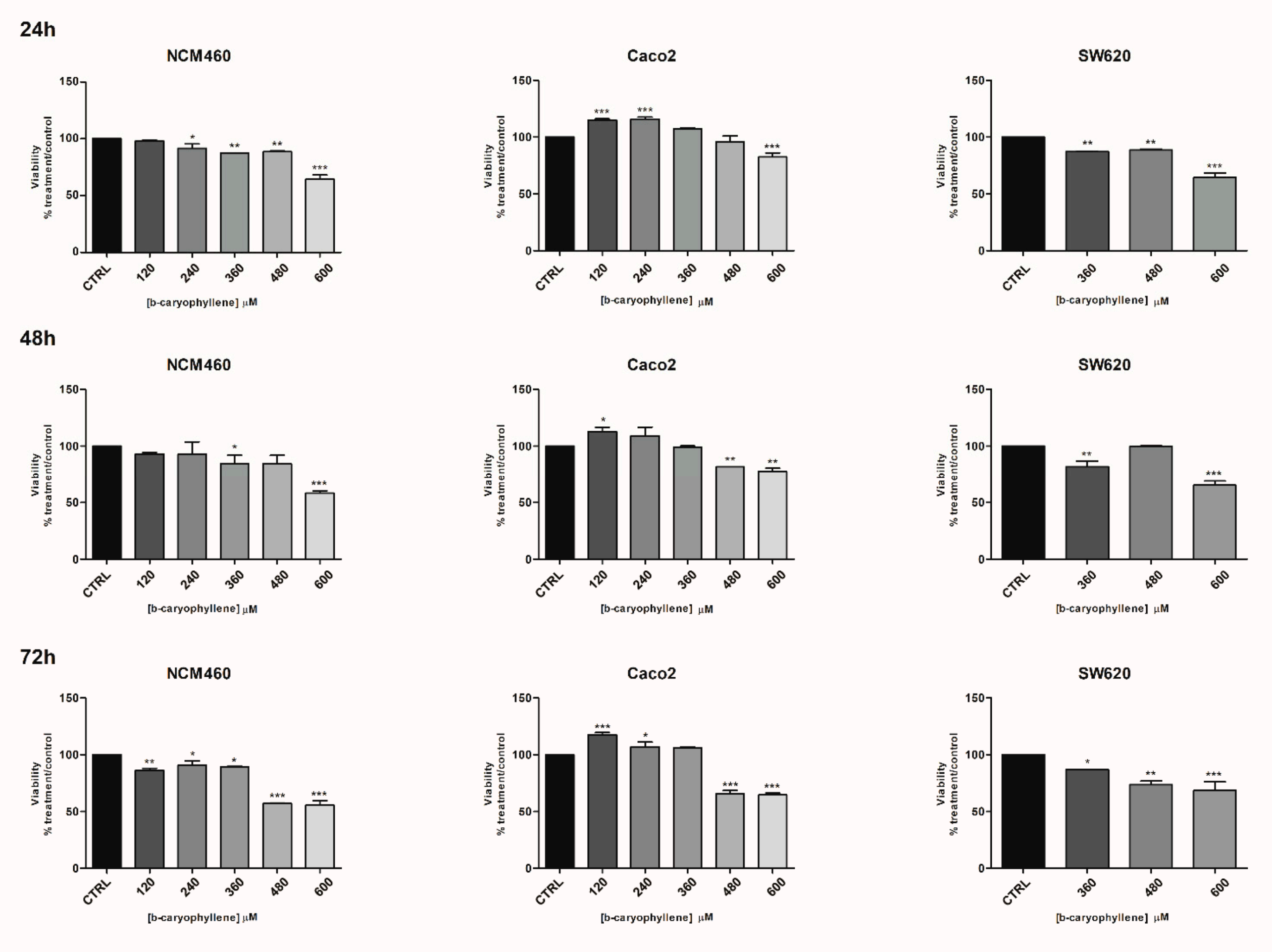

Figure S3. Effects of β-caryophyllene on the viability of NCM460, Caco2 and SW620 cell lines at three time points: 24, 48, 72 hours. The columns show the average median ± SE of three replicate experiments with three samples analysed per replicate. Significant statistical differences were evaluated with one-way ANOVA, followed by Bonferroni's Multiple Comparison test was applied to compare the treatment and the control groups: *=$p<0.05$; **=$p<0.01$; ***=$p<0.001$. NCM460: human normal colon mucosal epithelial cells; Caco2: human epithelial colorectal adenocarcinoma cells; SW620: colon cancer cells derived from lymph node metastatic site.

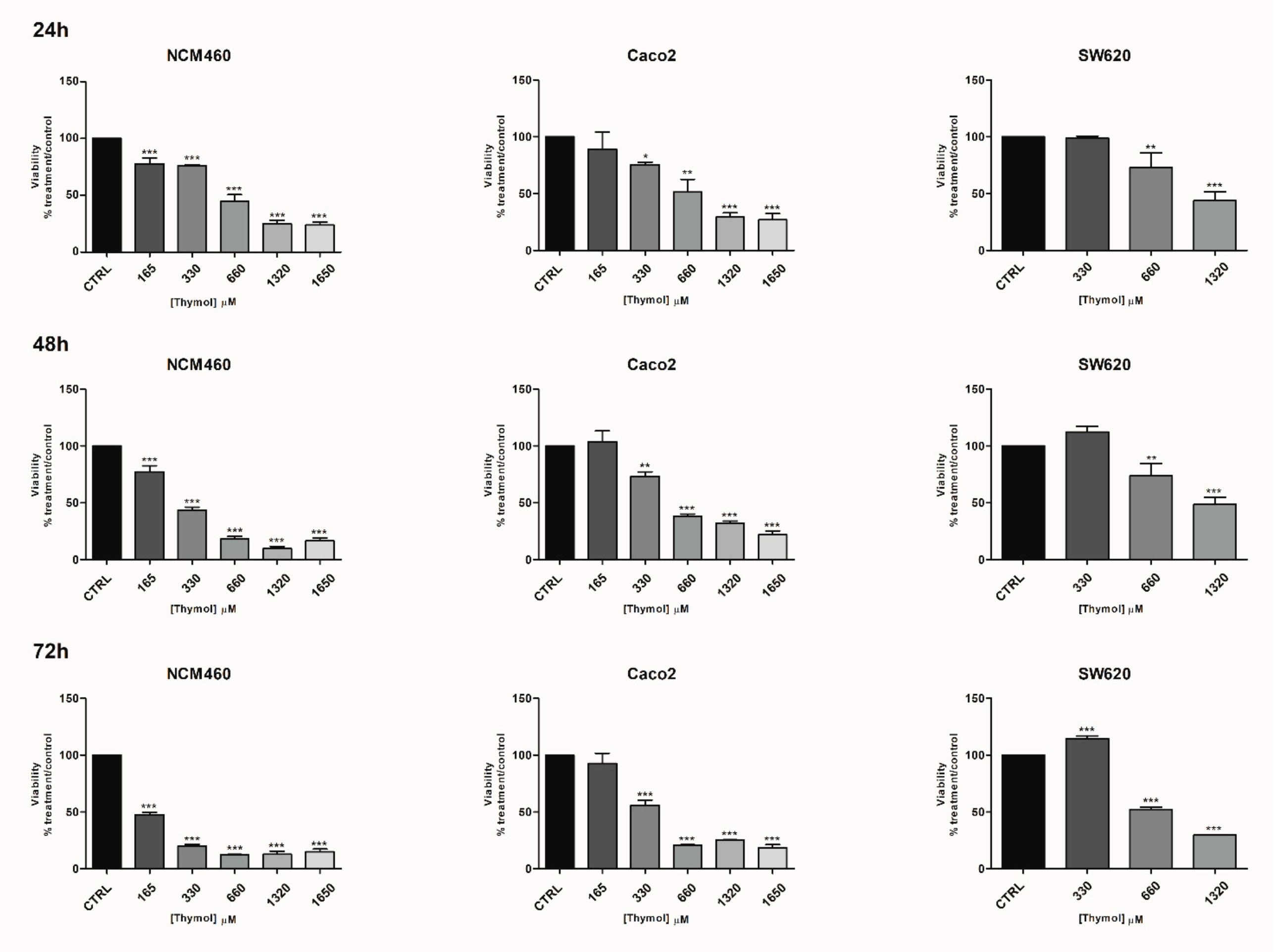

Figure S4. Effects of thymol on the viability of NCM460, Caco2 and SW620 cell lines at three time points: 24, 48, 72 hours. The columns show the average median ± SE of three replicate experiments with three samples analysed per replicate. Significant statistical differences were evaluated with one-way ANOVA, followed by Bonferroni's Multiple Comparison test was applied to compare the treatment and the control groups: *=p<0.05; **=p<0.01; ***=p<0.001. NCM460: human normal colon mucosal epithelial cells; Caco2: human epithelial colorectal adenocarcinoma cells; SW620: colon cancer cells derived from lymph node metastatic site.

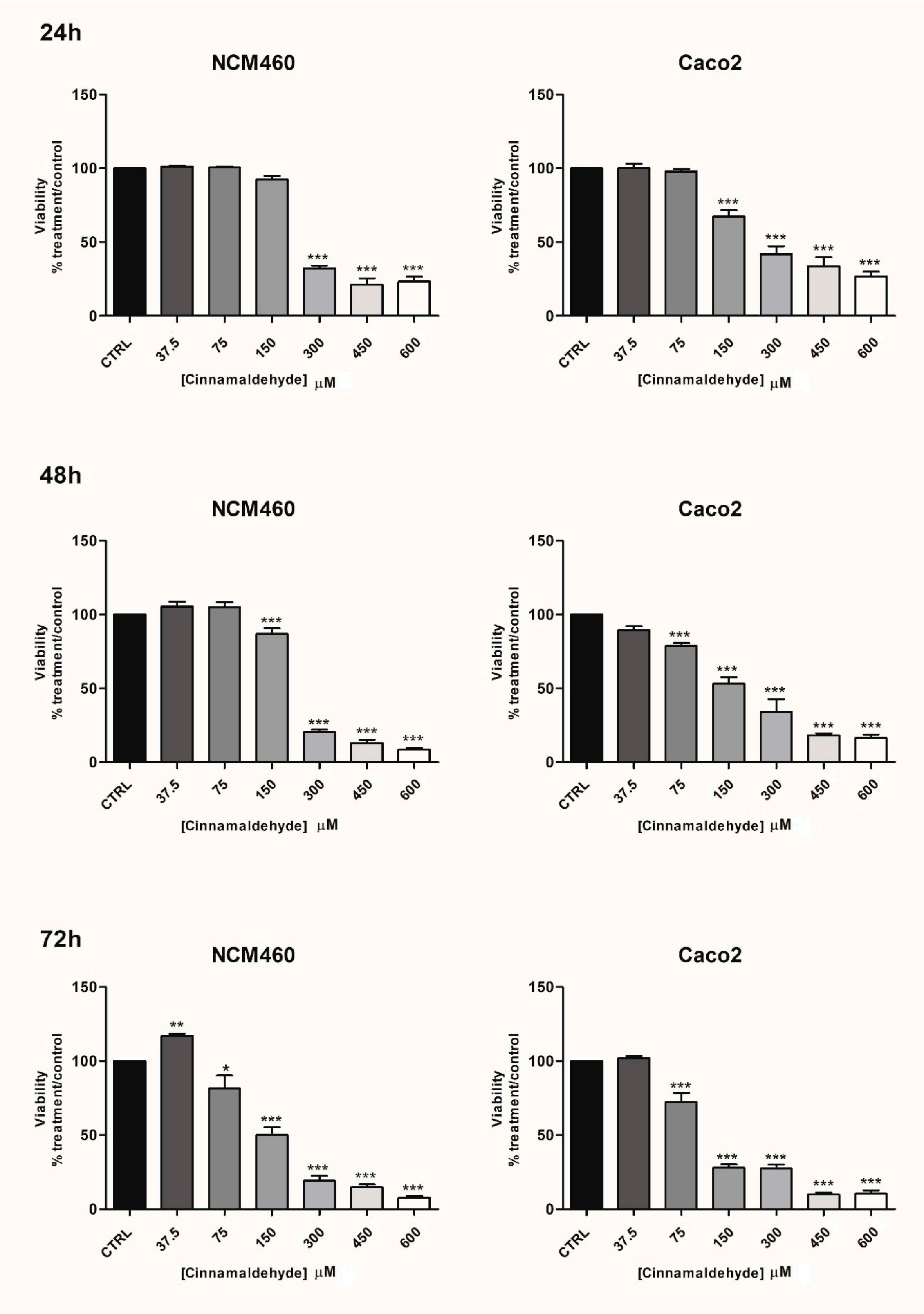

Figure S5. Effects of cinnamaldehyde on the viability of NCM460 and Caco2 cell lines at three time points: 24, 48, 72 hours. The columns show the average median ± SE of three replicate experiments with three samples analysed per replicate. Significant statistical differences were evaluated with one-way ANOVA, followed by Bonferroni's Multiple Comparison test was applied to compare the treatment and the control groups: *=p<0.05; **=p<0.01; ***=p<0.001. NCM460: human normal colon mucosal epithelial cells; Caco2: human epithelial colorectal adenocarcinoma cells.

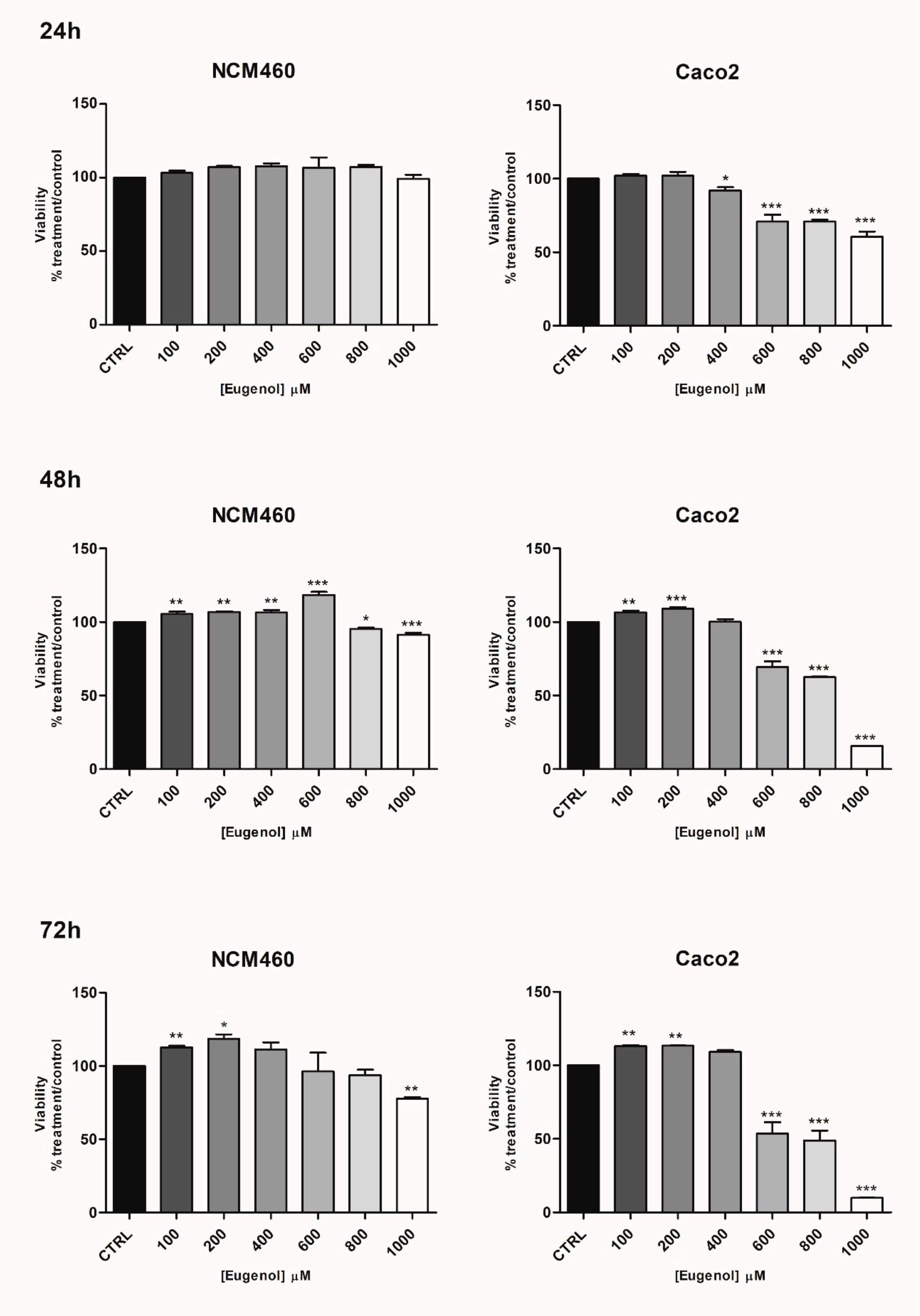

Figure S6. Effects of eugenol on the viability of NCM460 and Caco2 cell lines at three time points: 24, 48, 72 hours. The columns show the average median ± SE of three replicate experiments with three samples analysed per replicate. Significant statistical differences were evaluated with one-way ANOVA, followed by Bonferroni's Multiple Comparison test was applied to compare the treatment and the control groups: *=p<0.05; **=p<0.01; ***=p<0.001. NCM460: human normal colon mucosal epithelial cells; Caco2: human epithelial colorectal adenocarcinoma cells.
